# J. A. Pocock

**Published:** 1969-07

**Authors:** 


### OBITUARY

### J. A. POCOCK

Mr. J. A. Pocock died on 25th September, 1968, after a long and trying illness patiently borne. He was aged 63 and had been consultant surgeon to the United Bristol Hospitals and to Southmead Hospital. He was also Lecturer in Surgery in the Faculty of Medicine, University of Bristol. These cold facts do not do justice to a life which was devoted to both patients and students alike, spent in Bristol from 1937 onwards with the exception of the second world war period when he served in the R.A.F.

John Pocock was born on the 22nd August, 1905. His father was a distinguished general practitioner and his mother played a large part in the foundation of nursing in London. He went to school at Oundle and received his medical education at Cambridge and University College Hospital, London. He graduated M.B., B.Ch. in 1931 and passed the Final Fellowship Examination of the Royal College of Surgeons of England in 1934. He came to Bristol in 1937 when he was appointed Senior Resident Officer to the Bristol General Hospital. Some memories of those days are related by one who worked under John Pocock and who knew him intimately until his death. " John quickly established himself as a sound clinician and surgeon and won the affection of staff, nurses and patients. He created a most happy atmosphere with the rest of the resident staff at the B.G.H. He was always full of fun and was always coming out with amusing expressions. John's two joys in those days were a blue Alvis and his yacht. If we were fortunate we would be commandeered and would roar out of the hospital courtyard in the Alvis, to be rushed down to Portishead to help him sail round the dock ".

He was called up in 1939 and as a member of the Royal Air Force he served in many places during a busy and distinguished wartime career. In Egypt he was in charge of a surgical division and his ability was so recognised that he was asked to organise an R.A.F. Hospital Division in this country.

When hostilities ended he returned to Bristol to his former appointment and in 1946 he was appointed surgeon to the Bristol Royal Hospital. Of the B.G.H. days a Nurse writes—" He never failed to encourage and help and, when possible, to praise our work. If it was necessary he would commiserate with us. Likewise he never missed an opportunity of adding a little light relief to our day by filling up the glorious old Alvis with nurses and students and charging off with us. This meant more to us than he ever realised. The exceptional thing about John was that whether a titled person or a window cleaner, you were just as important. Acutely ill or unnecessarily anxious, he gave the whole of his attention, care and consideration."

When the National Health Service was introduced his appointment was transferred to the United Bristol Hospitals and to Southmead Hospital.

Ever since that time he played a full part in the surgical life of Bristol and the South West. He was a member of the Surgical Club of the South West of England and was its Honorary Treasurer for many years. He was especially interested in genito-urinary surgery and was a member of the British Association of Urological Surgeons. John Pocock was so interested in all aspects of surgery that his energies could not be confined to one speciality. He showed exceptional devotion to patients and was always intensely interested in them as people with problems which he must help to solve. He was so modest about his own clinical ability and technical skill that he was always willing to seek advice and was ready to consult will all his colleagues. His humility made him the friend of all and no trouble was too much for him if he thought that it would help either patient or colleague.

His devotion to patients was matched by his interest in all those with whom he worked Any of them would bear witness to help and advice freely given in such a kind and generous manner. The students and hospital doctors never failed to gain some benefit from contact with such a wonderful personality. The general practitioners of Bristol valued his opinion for the wisdom and care which he always gave so freely. He was never too busy to see a patient and he gave himself without thought for his own health and comfort to anyone who called on him for help.

John Pocock will be missed by all those who trained in Bristol during his lifetime. This personal loss will be exceeded by the large number of patients who were helped by him in sickness and in health. Many would wish to testify, but space does not permit. The following brief note is sent by a patient—" As two of the late Mr. Pocock's old patients, we would like to express a little of our appreciation of his skill as a surgeon, and his extreme kindliness of spirit. Mr. Pocock performed a series of severe operations on my sister 20 years ago, and she is now 87—a living testimony to his expertise and thoroughness. My own operation was of a minor nature, although painful, and I shall always remember the gentleness of his touch, and the personal interest he took in one's case."

Above all, his passing will mean the loss of a genuine interest in the practice of medicine in the widest sense. He was much concerned about the new horizons and was worried about the dangers and pitfalls in the brave new world. He felt that only if doctors remained humble and self effacing, could both the privacy of patients and the dignity of human suffering he preserved. For him the individual was always paramount and everything was subordinated to his central ideal. John was a strong character and he was always concerned lest the standards of the Bristol Medical School in particular and of medical practice in general should be lowered. He was kindness itself but he was not blind and his advice about people and things was sought because everyone knew that his opinion was both sound and unbiased.

Words cannot adequately describe a man of outstanding qualities. We all miss him, but none more than his wife, two sons and a daughter who had to share with him a large company of people during his lifetime.

